# Human ageing as a dynamic, emergent and malleable process: from disease-oriented to health-oriented approaches

**DOI:** 10.1007/s10522-019-09839-w

**Published:** 2019-10-08

**Authors:** Piotr Paweł Chmielewski

**Affiliations:** Division of Anatomy, Department of Human Morphology and Embryology, Faculty of Medicine, Wroclaw Medical University, 6a Chałubińskiego Street, 50-368 Wrocław, Poland

**Keywords:** Ageing, Disease, Homeodynamics, Longevity, Medical terminology, Senescence

## Abstract

Over the decades, biogerontology has matured as a scientific discipline. Currently, a number of theoretical frameworks are available to researchers when interpreting empirical data. Despite the great progress that has been made, a comprehensive understanding of biological processes that shape ageing is lacking. Senescence is a dynamic, plastic and highly complex metaphenomenon whose aetiology remains unclear. The paucity of information notwithstanding, some researchers promote ‘anti-ageing’ drugs and formulae every now and again. The rationale behind this concept is that ageing can be reduced to a mixture of biochemical reactions. Furthermore, the distinction between ageing and disease has been questioned on the grounds that ageing is the root of age-related diseases. It has been claimed that disease-oriented approaches can help delay ageing and prevent age-related diseases. Although these methods seem incongruous from an evolutionary standpoint, they become popular amongst the public. Moreover, if ageing is classified as a disease, this situation is likely to be exacerbated. Therefore, it is important to recognise the limitations of these reductionist and disease-oriented approaches. Only holistic and evidence-based strategies might be useful in slowing down ageing and preventing age-related diseases in the future.

## Introduction

Human ageing is a topic that has great practical importance because a large and increasing number of countries across the world must tackle the problem of ‘greying populations’ (Christensen et al. 2009; Le Bourg 2012; Olshansky and Carnes 2019). Furthermore, understanding of the ultimate and proximate causes of senescence is one of the key challenges in current biology and medicine (Kirkwood 2005; Rattan 2006; Reichard 2017). Although ageing research has burgeoned over the years and biogerontology has matured as a scientific discipline (Holliday 2006; Hayflick 2007a; Rattan 2012, 2014a, 2018), researchers do not agree on fundamental issues such as: (1) definitions of basic terms, e.g. ageing and longevity, (2) the nine or ten ‘hallmarks’ or seven ‘pillars’, (3) the distinction between ageing and disease, (4) the programmed/non-programmed nature of ageing within the life history and (5) effective strategies for delaying ageing and preventing age-related pathologies.

Meanwhile, it has been proposed that ageing can be classified as a disease (Bulterijs et al. 2015). Accordingly, many researchers promote ‘anti-ageing’ drugs or formulae based on the conviction that ageing is ‘easily treatable’. In my view, ageing as a highly complex metaphenomenon, i.e. a mixture of complex processes (Carnes et al. 2008), cannot be treated by reductionist or disease-oriented approaches. The causes of ageing are not well known. A comprehensive understanding of biological processes that shape senescence is lacking (Bonsall 2006; Hayflick 2016; Steiner et al. 2019). Therefore, the aetiology of ageing should be carefully explored before using ‘anti-ageing’ interventions. Only holistic approaches and evidence-based strategies might be useful in slowing down ageing and preventing age-related diseases in the future.

## Ageing as a dynamic and plastic metaphenomenon

According to traditional belief, human ageing is a passive, stochastic and inevitable phenomenon that is caused by a continuous accumulation of detrimental changes in tissues and organs, leading to a constantly increasing risk of death (Maddox 2001). From an evolutionary standpoint, senescence can be attributed to a failure of selection due to either pleiotropic constraints or declining strength of selection after reproductive maturity (Nelson and Masel 2017). From a mechanistic perspective, the ageing process is driven by the accumulation of random molecular damage (Kirkwood 2005) or molecular entropy (Hayflick 2007a, b). A corollary is that any simple method, such as calorie restriction (CR), a medication, a single gene mutation etc., is highly unlikely to slow down ageing in humans and other primates (Demetrius 2005; Le Bourg 2006; Shanley and Kirkwood 2006; Sikora et al. 2009). Even in future generations, most people will die before age 100 (Carnes et al. 2013).

Nevertheless, empirical data show that ageing can be viewed as a consequence of regulatory mechanisms for growth and development that actively control how cells, tissues and organs respond to their environment (Rizzo et al. 2017). It has been well established that the genes with major effects on longevity also regulate central aspects of metabolism (Warner et al. 2010). Moreover, data from genomics and proteomics suggest that there are specific signatures and ‘instructions’ for ageing at the molecular level (de Magalhães 2012). Briefly, multiple genes and microRNA (miRNA) are differentially expressed during progressive and regressive stages of ontogeny (Lui et al. 2010; Somel et al. 2010), which suggests that these age-related changes in gene expression and overarching epigenetic alternations originate in a developmental programme and are predetermined or ‘prearranged’ by this programme (Lenart and Bienertová-Vašku 2017). Furthermore, it has been demonstrated that partial reprogramming erases cellular markers of ageing in mouse and human cells (Ocampo et al. 2016). On balance, these findings seem inconsistent with the notion that there are no underlying mechanisms that actively control ageing. The contemporary understanding of ageing redefines the role of regulatory mechanisms, thereby suggesting that ageing is a dynamic and plastic metaphenomenon (Kenyon 2010, 2011; Kennedy and Lamming 2016; Ocampo et al. 2016). Recently, it has been hypothesised that an epigenetic clock controls the transition from the state of high somatic maintenance and normal bodily functioning to the state of low somatic maintenance and increasingly abnormal functioning (Mitteldorf 2015, 2016). Interestingly, if ageing and life are intertwined phenomena, as there are intimate relationships between ageing and metabolism, then it is questionable whether mutations that disrupt the ageing programme can produce non-ageing mutants that are immortal (cf. Kirkwood 2005; Chmielewski 2017). Nevertheless, the discussion concerning the ‘great conundrum’ in biogerontology has reached deadlock (cf. Kirkwood and Melov 2011; Kowald and Kirkwood 2016; Skulachev and Skulachev 2017; Mitteldorf 2017, 2018).

Currently, the hallmarks and pillars of ageing are intensively studied in a wide variety of research areas (López-Otín et al. 2013; Kennedy et al. 2014). It has been suggested that targeting these mechanisms directly can help delay ageing and prevent age-related pathologies in the future. If ageing is a dynamic and plastic process, rational strategies and interventions can effectively postpone senescence. Nonetheless, the peculiarity of ageing consists in its complexity (Chmielewski et al. 2016; Cohen 2016; Arking 2019), which explains why there is no cure for ageing. Therefore, some researchers argue that answering the question ‘what is ageing?’ is tantamount to answering the question ‘what is life?’ (da Costa et al. 2016).

## Slowing down ageing: from disease-oriented to health-oriented approaches

There are two conceptualisations of age-related frailty, i.e. a condition of increased vulnerability to stresses and diseases in late ontogeny, often accompanied by chronic fatigue, physical weakness and decline in activity. According to the standard medical model, the accumulation of various deficits with age drives this state of increased susceptibility to diseases. This line of reasoning is consistent with the paradigm’s focus on the way in which medical interventions can affect health and longevity. According to the standard biological model, frailty is caused by the accumulation of molecular damage that is also responsible for ageing. In this view, frailty can develop independently from any disease. This model is associated with health-oriented approaches and pro-longevity interventions.

In accordance with a long-standing tradition in medicine, all diseases have been targeted separately, neglecting the links between organ systems in the body. Fortunately, science and medicine have made great advances in the understanding of these links (Kennedy et al. 2014). Nevertheless, disease-oriented approaches deal with the final stages of debilitating processes, neglecting strategies for the enhancement of the homeodynamic capabilities of the body. Moreover, the implementation of reductionist and disease-oriented approaches in the field of ageing, but especially the use of ‘anti-ageing’ drugs, is scientifically ungrounded. From an evolutionary standpoint, such methods seem incongruous. This line of reasoning is consistent with the fact that ageing is not a monolithic and reducible process but a highly complex metaphenomenon that arises as a by-product from life history adaptations (Holliday 2007; Rattan 2014a; Cohen 2016; Chmielewski 2017).

Recently, it has been suggested that it will be advantageous to classify ageing as a disease (Bulterijs et al. 2015). Although some researchers argue that the distinction between normal ageing (‘normal dysfunction’) and pathologies (‘abnormal dysfunction’) is not completely clear and should be abandoned (Izaks and Westendorp 2003; Gems 2015; Stambler 2015, 2017), this goes against a long tradition. Sound arguments have been adduced to explain that ageing can never be understood as a disease (Rattan 2013, 2014b, 2016; Hayflick 2016; Chmielewski 2018). Furthermore, if ageing is classified as a disease, then the links between ageing and the wide spectrum of age-related pathologies might become a neglected area of research.

Every now and then, some authors promote ‘anti-ageing’ drugs or formulae based on reductionist and disease-oriented approaches (e.g. Blagosklonny 2017, 2018). The rationale behind this concept is that ageing can be reduced to a mixture of biochemical reactions (Diaconeasa et al. 2015). It is questionable whether these approaches are applicable to ageing and whether these ‘anti-ageing’ drugs can enhance healthspan and longevity. Whilst suitable exercise, a proper diet, a positive attitude and a network of close friends have well-established health benefits, the use of ‘anti-ageing’ drugs has a number of limitations: (1) treatment of disease can delay the age of death but it cannot slow down ageing defined as a stochastic process resulting from the escalating loss of molecular fidelity that ultimately exceeds repair capacity (Hayflick 2004, 2007a, b; Carnes et al. 2008), (2) drugs cannot enhance the homeodynamic capabilities of the body in the long run (Cohen 2016), (3) they should not be used by healthy people because: (a) the long-term effects of these ‘anti-ageing’ drugs are unknown, (b) counterindications include drug allergies and exercise-induced food allergies, (c) the use of drugs might negatively affect patients’ psychological status, (d) these approaches and methods are precarious as some people might discontinue a healthy lifestyle, (e) there are also ethical issues regarding such interventions, (4) they cannot be used by patients who use other drugs because drugs would interact with each other, (5) in these reductionist and disease-oriented approaches, inter-individual variability is neglected, and ageing is so complex that it is reasonable to predict that every person should be treated differently (e.g. is the use of metformin equally justified in a hyperglycaemic person and in a hypoglycaemic person?).

The long-term effects of these ‘anti-ageing’ drugs might be harmful. For example, well-known side-effects of propranolol include bradycardia, fatigue, bronchial constriction and metabolic changes. Also the most powerful non-pharmacological intervention that can slow down ageing in model animals, i.e. CR, is associated with side-effects such as psychological disturbances, behavioural changes and metabolic alterations (Redman et al. 2009). Unlike physical exercise, CR reduces bone mass, muscle mass, muscle size and strength and maximal aerobic capacity in proportion to the reduction in body weight (Villareal et al. 2006; Weiss et al. 2007). Furthermore, there is evidence that CR is associated with impaired glucose tolerance in subsequent generations (Zambrano et al. 2005; Pinheiro et al. 2008). Therefore, the use of naturally occurring compounds in the fight against ageing seems more judicious (Sikora et al. 2010; Gómez-Linton et al. 2019).

Nevertheless, only holistic approaches along with sophisticated tools and methods, such as bioinformatics and systems biology, can further our understanding of the nature of ageing. Biogerontology might profit from focusing on the health-oriented approaches and pro-longevity interventions (Arking 2019). These strategies can improve the quality of life in older people. Concurrently, they can reduce the economic burdens of a burgeoning ageing population hampered by multiple chronic diseases (Kennedy et al. 2014).
